# K_ATP_ channels modulate intrinsic firing activity of immature entorhinal cortex layer III neurons

**DOI:** 10.3389/fncel.2014.00255

**Published:** 2014-08-27

**Authors:** Maria S. Lemak, Oksana Voloshanenko, Andreas Draguhn, Alexei V. Egorov

**Affiliations:** ^1^Institute of Physiology and Pathophysiology, Heidelberg UniversityHeidelberg, Germany; ^2^Institute of Higher Nervous Activity and Neurophysiology, Russian Academy of SciencesMoscow, Russia; ^3^Division of Signalling and Functional Genomics, German Cancer Research CenterHeidelberg, Germany; ^4^Bernstein Center for Computational Neuroscience Heidelberg/MannheimHeidelberg, Germany

**Keywords:** prolonged intrinsic bursts, network, K_ATP_ channels, entorhinal cortex, layer III, slice, postnatal rat

## Abstract

Medial temporal lobe structures are essential for memory formation which is associated with coherent network oscillations. During ontogenesis, these highly organized patterns develop from distinct, less synchronized forms of network activity. This maturation process goes along with marked changes in intrinsic firing patterns of individual neurons. One critical factor determining neuronal excitability is activity of ATP-sensitive K^+^ channels (K_ATP_ channels) which coupled electrical activity to metabolic state. Here, we examined the role of K_ATP_ channels for intrinsic firing patterns and emerging network activity in the immature medial entorhinal cortex (mEC) of rats. Western blot analysis of Kir6.2 (a subunit of the K_ATP_ channel) confirmed expression of this protein in the immature entorhinal cortex. Neuronal activity was monitored by field potential (fp) and whole-cell recordings from layer III (LIII) of the mEC in horizontal brain slices obtained at postnatal day (P) 6–13. Spontaneous fp-bursts were suppressed by the K_ATP_ channel opener diazoxide and prolonged after blockade of K_ATP_ channels by glibenclamide. Immature mEC LIII principal neurons displayed two dominant intrinsic firing patterns, prolonged bursts or regular firing activity, respectively. Burst discharges were suppressed by the K_ATP_ channel openers diazoxide and NN414, and enhanced by the K_ATP_ channel blockers tolbutamide and glibenclamide. Activity of regularly firing neurons was modulated in a frequency-dependent manner: the diazoxide-mediated reduction of firing correlated negatively with basal frequency, while the tolbutamide-mediated increase of firing showed a positive correlation. These data are in line with an activity-dependent regulation of K_ATP_ channel activity. Together, K_ATP_ channels exert powerful modulation of intrinsic firing patterns and network activity in the immature mEC.

## INTRODUCTION

Neuronal networks express highly organized multi-neuronal activity patterns which are believed to mediate specific behavioral and cognitive functions. During ontogenesis, coherent patterns develop from distinct, less synchronized forms of network activity (Khazipov and Luhmann, 2006; Egorov and Draguhn, 2013). These immature patterns, on the other hand, might be involved in brain maturation, including neuronal growth, synapse formation and network wiring (Ben-Ari, 2002; Kasyanov et al., 2004; Mohajerani and Cherubini, 2006). Early network patterns are highly diverse depending on brain structures and developmental stages (Feller et al., 1996; Leinekugel et al., 1997; Kandler and Katz, 1998; Dupont et al., 2006; Crepel et al., 2007; Allène et al., 2008). Distinct mechanisms underlying immature activity patterns include electrical coupling between neurons, excitatory actions of GABA, synchronous activation of glutamatergic synapses as well as intrinsic neuronal bursting (Garaschuk et al., 2000; Sipilä et al., 2005; Zheng et al., 2006; Crepel et al., 2007; Allène et al., 2008; Sheroziya et al., 2009).

Neuronal excitability strongly depends on cellular energy metabolism. Lack of energy is particularly damaging during the prenatal and early postnatal periods, resulting in lasting neurological deficits throughout life (Nelson, 1989; Erecinska et al., 2004). ATP-sensitive K^+^ channels (K_ATP_ channels) provide a unique link between cellular energy state and electrical activity. K_ATP_ channels are inwardly rectifying K^+^-selective ion channels that are inhibited by intracellular ATP. A decrease of submembrane ATP levels and accompanying rise in ADP triggers K_ATP_ channel opening (Seino, 1999; Haller et al., 2001). K_ATP_ channels exist in many excitable cells, including cardiac myocytes, skeletal muscle cells, pancreatic β-cells (Ashcroft and Ashcroft, 1990) and neurons (Karschin et al., 1997; Dunn-Meynell et al., 1998; Zawar et al., 1999). In excitable tissues, these channels act as metabolically controlled “excitation brakes” by hyperpolarizing cells in conditions of low ATP supply. K_ATP_ channels are composed of four pore-forming Kir6 subunits, and four regulatory sulfonylurea receptor (SUR) subunits (Aguilar-Bryan and Bryan, 1999; Nichols, 2006). While K_ATP_ channels appear to play an important role in protecting neurons against ischemic or anoxic injury (Fujimura et al., 1997; Yamada et al., 2001; Sun et al., 2007) they are also activated in normal network states, e.g., during burst firing in respiratory neurons (Haller et al., 2001). In hippocampal granule cells open probability of single K_ATP_ channels transiently increases in response to modest firing activity (Tanner et al., 2011).

The entorhinal cortex (EC) constitutes the major interface between the hippocampus and parahippocampal areas and plays a crucial role in spatial cognition and memory processing (Squire et al., 2004; van Strien et al., 2009; Buzsáki and Moser, 2013). Principal neurons of EC layer III (LIII) provide direct input to the apical dendritic tuft of hippocampal CA1 pyramids (Witter and Amaral, 2004) which is an important pathway for temporal association memory and fear learning (Suh et al., 2011; Kitamura et al., 2014; Lovett-Barron et al., 2014). In adult rats, medial EC (mEC) LIII principal neurons are regularly firing cells that do not discharge in bursts (Dickson et al., 1997; Gloveli et al., 1997; Yoshida and Alonso, 2007). In contrast, during early postnatal maturation a fraction of mEC LIII principal neurons spontaneously generates prolonged Ca^2+^ – and voltage-dependent intrinsic bursting activity (Sheroziya et al., 2009). These burst discharges involve the Ca^2+^-sensitive non-specific cationic current (I_CAN_), persistent Na^+^ current (I_Nap_), and – for termination – Ca^2+^-activated K^+^ current (I_AHP_; Sheroziya et al., 2009).

Given that increased firing frequency or bursts can elicit opening of K_ATP_ channels (Haller et al., 2001; Tanner et al., 2011), we investigated the role of K_ATP_ channels for cellular and network activity in the immature mEC.

We report that excitability of immature neurons and early patterns of network oscillations are powerfully modulated via K_ATP_ channels. These findings indicate that neuronal ATP consumption and energy demand might have important consequences for postnatal activity-dependent maturation of neurons and networks in the mEC.

## MATERIALS AND METHODS

### ETHICAL APPROVAL

All experimental protocols were performed in accordance with the National Institutes of Health Guide for the Care and Use of Laboratory Animals and were approved by the ethical committee of the Institute of Higher Nervous Activity and Neurophysiology, Russian Academy of Sciences (IHNA RAS) or by the state government of Baden-Württemberg, Germany. All efforts were made to minimize animal suffering and to reduce the number of animals used.

### PREPARATION OF BRAIN SLICES

Horizontal brain slices (350–600 μm thick) containing the hippocampus, entorhinal and parts of perirhinal cortices were obtained from Wistar rats at postnatal day (P) 6–13 using standard procedures. P0 was taken as the day of birth. Rats were purchased from Charles River Laboratories (Sulzfeld, Germany) or from the local veterinary service (INHA RAS, Russia). Animals were decapitated, brains were rapidly removed and placed in cold (1–4°C) oxygenated artificial cerebrospinal fluid (ACSF) containing (in mM): 124 NaCl, 3 KCl, 1.6 CaCl_2_, 1.8 MgSO_4_, 26 NaHCO_3_, 1.25 NaH_2_PO_4_, and 10 glucose (for recordings in interface-type chambers) or 130 NaCl, 3.5 KCl, 1.2 NaH_2_PO_4_, 25 NaHCO_3_, 1.3 MgCl_2_, 1 or 2 CaCl_2_, and 25 glucose (for recordings in submerged conditions). Solutions were saturated with 95% O_2_ and 5% CO_2_ (pH 7.4 at 37°C). Brain slices were cut using a Vibratome (Leica VT1000S, Germany or Campden Instruments, Loughborough, UK). For extracellular fp recordings, slices were transferred into a Haas-type interface chamber, maintained at 34 ± 1°C and superfused with ACSF at a rate of 1.5–2 ml/min. Prior to electrophysiological recordings, slices were allowed to recover for at least two hours. For whole-cell patch-clamp recordings under submerged conditions, slices were stored at 34 ± 1°C for 10 min in a holding bath containing ACSF, before cooling down to room temperature. After incubation for at least 1 h at room temperature, individual slices were transferred into a recording chamber, superfused with oxygenated ACSF at a rate of 3–6 ml/min and maintained at 33 ± 1°C.

### RECORDING PROCEDURES

Whole-cell patch-clamp recordings were performed under visual guidance using an Olympus microscope fitted with infrared differential interference contrast optics (Olympus BX51WI). We preferentially recorded pyramidal neurons located in the deep part of LIII in order to exclude potential recording from LII pyramidal cells. The lamina dissecans, a distinct cell-free zone (sometimes referred to as layer IV), was used as a reference to identify the border between LIII and layer V. Current-clamp recordings were performed with an Axopatch 1D patch-clamp amplifier (Axon Instruments, Foster City, CA, USA) or ELC-03XS amplifier (npi electronics, Tamm, Germany). Patch electrodes were backfilled with the following solution (in mM): 115 K-gluconate, 20 KCl, 10 disodium phosphocreatine, 10 HEPES, 4 MgATP, and 0.3 GTP or 135 K-gluconate, 20 NaCl, 10 HEPES, 3.95 Mg-gluconate, 0.05 MgATP, and 0.3 GTP (tip resistance of 5–7 MΩ). The electrode solutions were adjusted to pH 7.3 with 1 M KOH. Data were low-pass filtered at 1–2 kHz, digitized at 5–10 kHz (Digidata 1322A, Molecular Devices), and stored on a personal computer using the AxoScope software package (Molecular Devices). Extracellular fp recordings were performed with an EXT 10-2F amplifier (npi electronics, Tamm, Germany). Signals were amplified 100×, low-pass filtered at 2 kHz and high-pass filtered at 0.3 Hz, digitized at 20 kHz with an analog-to-digital converter (Cambridge Electronic Design (CED) MICRO 1401 mkll, Cambridge, UK) and saved on a computer using Spike2 software (CED, Cambridge, UK) for oﬄine analysis. Fp recordings were obtained with ACSF-filled borosilicate glass electrodes (tip diameter 3–5 μm) placed in LIII of the mEC. In the interface chamber, we identified the EC and its layers with a dissecting microscope.

### CHEMICALS

Tolbutamide (300 μM), glibenclamide (1–10 μM) and diazoxide (100–400 μM) were obtained from Sigma-Aldrich (Taufkirchen, Germany) and NN414 (5–25 μM) was ordered from Axon Medchem (Groningen, Netherlands). All drugs were bath-applied at the desired concentrations from stock solutions made in DMSO. The final concentration of DMSO in ACSF was ≤0.1%. Control experiments revealed no measurable effects of DMSO on cellular properties or network events (*n* = 4). Block of ionotropic glutamate- and GABA_A_-receptor mediated neurotransmission was performed with a cocktail of kynurenic acid (2 mM) and picrotoxin (100 μM) obtained from Sigma-Aldrich (Taufkirchen, Germany).

### WESTERN-BLOTS ANALYSIS

For Western-blots analysis horizontal brain slices (600–700 μm thick) were obtained from Wistar rats at P8 and P13 using standard procedures as described above. Areas of interest (hippocampal CA1 and EC, including lateral and medial areas) were dissected from individual slices under visual control, placed into Eppendorf tubes, and stored at -20°C. Then, tissue was treated with Lysis Buffer (20 mM Tris–HCl pH 7.4, 130 mM NaCl, 10% Glycerol (w/v), 2 mM EDTA), supplemented with 1% Triton-X-100 and complete mini protease inhibitor (Roche) according to manufacturer’s instructions. Lysates were separated on NuPage 4–12% Gels (Invitrogen) using MOPS buffer, and blotted on Hybond ECL membranes (GE Healthcare). Separation of proteins was checked using Ponceau S solution. We then applied the Qentix Western Blot Signal Enhancer (Perboi/Thermo Fisher, #21050) according to the protocol, and then blocked the membrane with 5% milk for 1 h before incubation with primary rabbit anti-Kir6.2 polyclonal antibodies (1:10,000; AB5495, Millipore, Temecula, USA) at 4°C overnight. Secondary anti-rabbit antibodies (VWR, NA934) were applied for 1 h at 1:10,000 dilution. The membrane was stripped and blotted with actin antibodies (1:20,000; Abcam, AC-15, ab6276) for 1 h and then incubated with secondary anti-mouse (VWR, NA931) for 1 h. Specificity of Kir6.2 antibody was tested by siRNA silencing of this gene in HEK293T cells (DMEM, Invitrogene, +10% FBS). HEK293T cells were reverse transfected with RNAImax (Invitrogene, 13778150) and 5 nM of KCNJ11/Kir6.2 (Ambion/Life technologies, s7761 CCTGTACTGGGTTATTTTT) or negative control#1 (Ambion/Life technologies) for 72 h. Knock-down efficiency of more than 75% was controlled by RT-qPCR. RNA was isolated with the help of RNeasy Mini Kit (Qiagen), cDNA was synthetized using RevertAid H Minus First Strand cDNA kit (Thermo Fisher Scientific) and qPCR was performed using Roch Applied Science Universary probe system with forward 5′-agcagtgttgtgtgaacttgc-3′, reverse 5′-cagcaagaaaagcccagagt-3′ primers and probe#8 and UBC as housekeeping gene control (forward 5′-ctgatcagcagaggttgatcttt-3′, reverse 5′-tctggatgttgtagtcagacagg-3′ and probe #11).

### IMMUNOHISTOCHEMISTRY

For fluorescence staining, 450 μm thick slices were fixed in 4% paraformaldehyde (PFA) in phosphate buffer (PB) for at least 12 h (4°C). They were then embedded in 4% agar, re-sliced at 70 μm thickness (VT 1000S, Leica, Germany), mounted on superfrost plus microscope slides (Menzel-Gläser, Braunschweig, Germany) and stored at -20°C. For staining, slices were permeated in methanol for 10 min at -20°C, rehydrated in phosphate buffered saline (PBS, room temperature) and incubated for 10 min in 0.3 M glycine in PBS to minimize background fluorescence. Slices were then pretreated for 1 h in blocking buffer (10% goat serum, 1% Triton X-100 in PBS). Antibodies were diluted in antibody solution (1% goat serum, 0.3% Triton X-100 in PBS) and incubated overnight (>16 h) for primary antibodies and 2 h for secondary antibodies at room temperature. Rabbit anti-Kir6.2 primary antibodies (1:500; AB5495, Millipore, Temecula, CA, USA) and secondary Alexa Fluor 488 IgG goat against rabbit (1:1000; Invitrogen, Eurgene, OR, USA) were used for immunochemical staining. DAPI (1:10,000; Invitrogen, Eugene, OR, USA), was used as nuclear stain to visualize topology.

### DATA ANALYSIS

Electrophysiological data were analyzed off-line using Spike2 software (CED, Cambridge, UK) and Clampfit (Molecular Devices). Fp activity was analyzed from primary data sections lasting at least 10 min. Duration of fp-bursts was measured from the onset of negative potential deflection until the peak of positivity before the field waveform return to baseline level. The amplitude of fp-bursts was calculated as the difference between baseline and the negative peak of the fp transient. Whole-cell recordings were typically started 10–15 min after break-in, when balance between intracellular millieue and patch-solution was established. When depolarizing current injections were needed to induce bursting, recording was delayed for at least 5 min after onset of stable bursting. Bursting neurons were recorded in standard bicarbonate-based ACSF containing 1 mM Ca^2+^, and regularly firing cells in ACSF containing 1 or 2 mM Ca^2+^. Spontaneous intrinsic bursting activity was analyzed from at least 3 min of recordings. Burst duration was calculated as the time between the first and the last spike within a burst. Regular firing was induced by depolarizing current steps (11 or 35 s duration, +9 to +80 pA), with intervals between pulses of 19 or 55 s, respectively. Regular firing frequency was analyzed from the 10 s of a depolarizing step (averaged data from three current pulses) under control conditions, in the presence of drugs and after washout. For statistical evaluation of drug effects, baseline values were compared to the latest phase of the interval with drugs present (at least 30 min for fp recordings/interface-type and 15 min for whole-cell recordings/submerged-type).

### STATISTICAL ANALYSIS

Averaged data are given as mean ± SEM. Statistical analysis was performed using GraphPad (InStat, San Diego, CA, USA) software. Fp parameters and part of single cell data were compared by a one-way repeated-measures ANOVA followed by appropriate *post hoc* tests, depending on parametric or non-parametric data distribution. Paired two-tailed Student’s *t*-test or Wilcoxon matched pairs signed ranks test was used for statistical comparison of the remaining single cell data. A *p* value <0.05 was regarded as significant. For all data: ^∗^*p* < 0.05, ^∗∗^*p* < 0.01, ^∗∗∗^*p* < 0.001, ns, not significant.

## RESULTS

**FIGURE 1:**
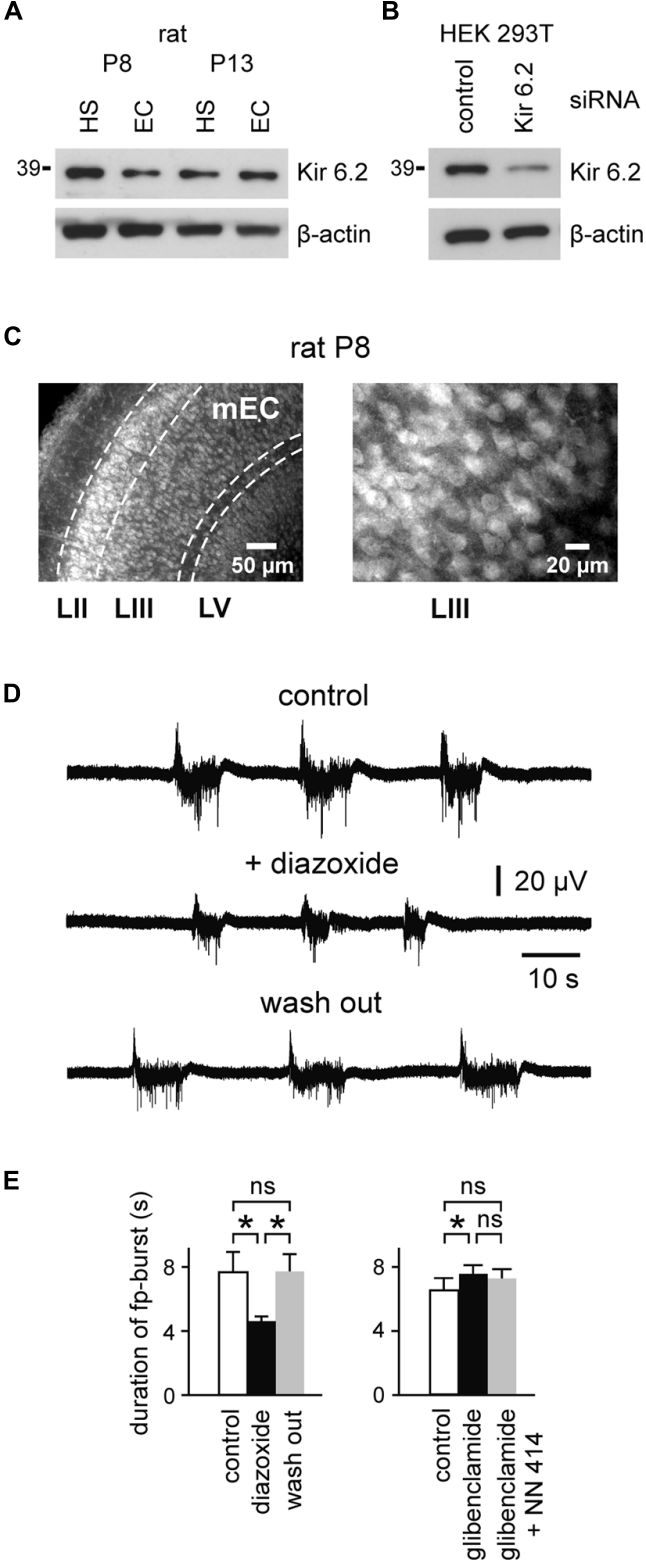
**Expression of the K_ATP_ channel-forming Kir6.2 subunits and modulation of spontaneous fp-bursts by K_ATP_ channels in the immature EC. (A)** Western blot analysis of Kir6.2 protein expression in the hippocampus (HS; area CA1) and entorhinal cortex (EC) of rats at P8 and P13. Results are representative for three independent experiments. **(B)** Determination of Kir6.2 antibody specificity. Western blots of HEK293T cell lysate after reverse transfection with control- or Kir6.2-siRNA, respectively, for 72 h. β-actin served as the loading control (bottom panels in **A,B**). **(C)** Micrographs of Kir6.2 immunoreactivity in the rat mEC at P8. Layers II, III, and V are indicated at the bottom. Right panel shows magnification of layer III. **(D)** Decrease of spontaneous field potential (fp) bursts by the K_ATP_ channel opener diazoxide (400 μM). **(E)** Left: effects of diazoxide on fp-burst duration (*n* = 8). Right: effect of the K_ATP_ channel blocker glibenclamide (10 μM) and further adding of the K_ATP_ channel opener NN414 (25 μM) on fp-burst duration (*n* = 14). Averaged data are given as mean ± SEM. ANOVA followed by Bonferroni’s *post hoc* test, *p < 0.05, ns, not significant.

We first analyzed expression of Kir6.2 (a major subunit of the K_ATP_ channel) within the second postnatal week. We found that Kir6.2 is extensively expressed in the EC at the protein level, suggesting the presence of K_ATP_ channels in this structure during an early developmental stage (**Figure 1A**). Specificity of antibodies was shown in HEK293T cells upon silencing of Kir6.2 expression by siRNA (**Figure 1B**; for details, see Materials and Methods). Immunocytochemical experiments revealed Kir6.2 expression across all layers of the immature rat mEC (**Figure 1C**). Kir6.2 immunoreactivity was predominately detected on somata and proximal dendrites of individual cells located in layers II, III, and V.

In order to address the functional role of K_ATP_ channels at such early stages, we investigated network- and single-cell activity in the mEC. Fp recordings within layer III of slices from rats at P10–P13 revealed spontaneous repetitive bursts. These glutamate receptor-mediated events were characterized by prolonged fp shifts (duration 6.9 ± 0.7 s, peak amplitude 0.056 ± 0.004 mV, *n* = 22 slices), superimposed by fp fluctuations at ∼15–30 Hz and multiple unit discharges, as previously reported by Sheroziya et al. (2009). Importantly, the K_ATP_ channel opener diazoxide (400 μM) reversibly reduced the duration of fp-bursts from 7.7 ± 1.3 s to 4.6 ± 0.3 s (*n* = 8, *p* < 0.05, ANOVA Bonferroni’s *post hoc*; **Figures 1D,E**). The K_ATP_ channel blocker glibenclamide (10 μM) had opposite effects and slightly prolonged fp-bursts (control vs. glibenclamide; 6.5 ± 0.8 s vs. 7.6 ± 0.5 s, *n* = 14, *p* < 0.05, ANOVA Bonferroni’s *post hoc*; **Figure 1E**). The effect of glibenclamide was not reversible after washout of the drug (tested in five slices). Indeed, fp-burst duration showed a further increase following washout of the substance. We therefore opted to antagonize the effect of the K_ATP_ channel blocker glibenclamide with the SUR1-specific K_ATP_ channel opener NN414 (Dabrowski et al., 2003; 25 μM). This led to a slight decrease of fp-burst duration which, however, did not reach significance (glibenclamide vs. NN414; 7.6 ± 0.5 vs. 7.2 ± 0.6 s, *n* = 14, *p* > 0.05, ANOVA Bonferroni’s *post hoc*; **Figure 1E**). Frequency of fp-bursts (i.e., burst occurrences per minute) was slightly reduced in the presence of diazoxide (control vs. diazoxide; 3.6 ± 0.4 vs. 2.4 ± 0.3, *p* < 0.05, ANOVA Bonferroni’s *post hoc*), and was not affected by glibenclamide (control vs. glibenclamide; 3.6 ± 0.4 vs. 3.7 ± 0.3, *p* > 0.05, ANOVA Bonferroni’s *post hoc*). Amplitude of fp-bursts was not significantly affected by both drugs (control vs. diazoxide; 0.061 ± 0.008 vs. 0.055 ± 0.005 mV, control vs. glibenclamide; 0.054 ± 0.005 vs. 0.05 ± 0.004 mV, *p* > 0.05 for both drugs, ANOVA Bonferroni’s *post hoc*). Together, these results suggest that activity of K_ATP_ channels regulates the generation and duration of spontaneous fp-bursts in the developing mEC.

**FIGURE 2:**
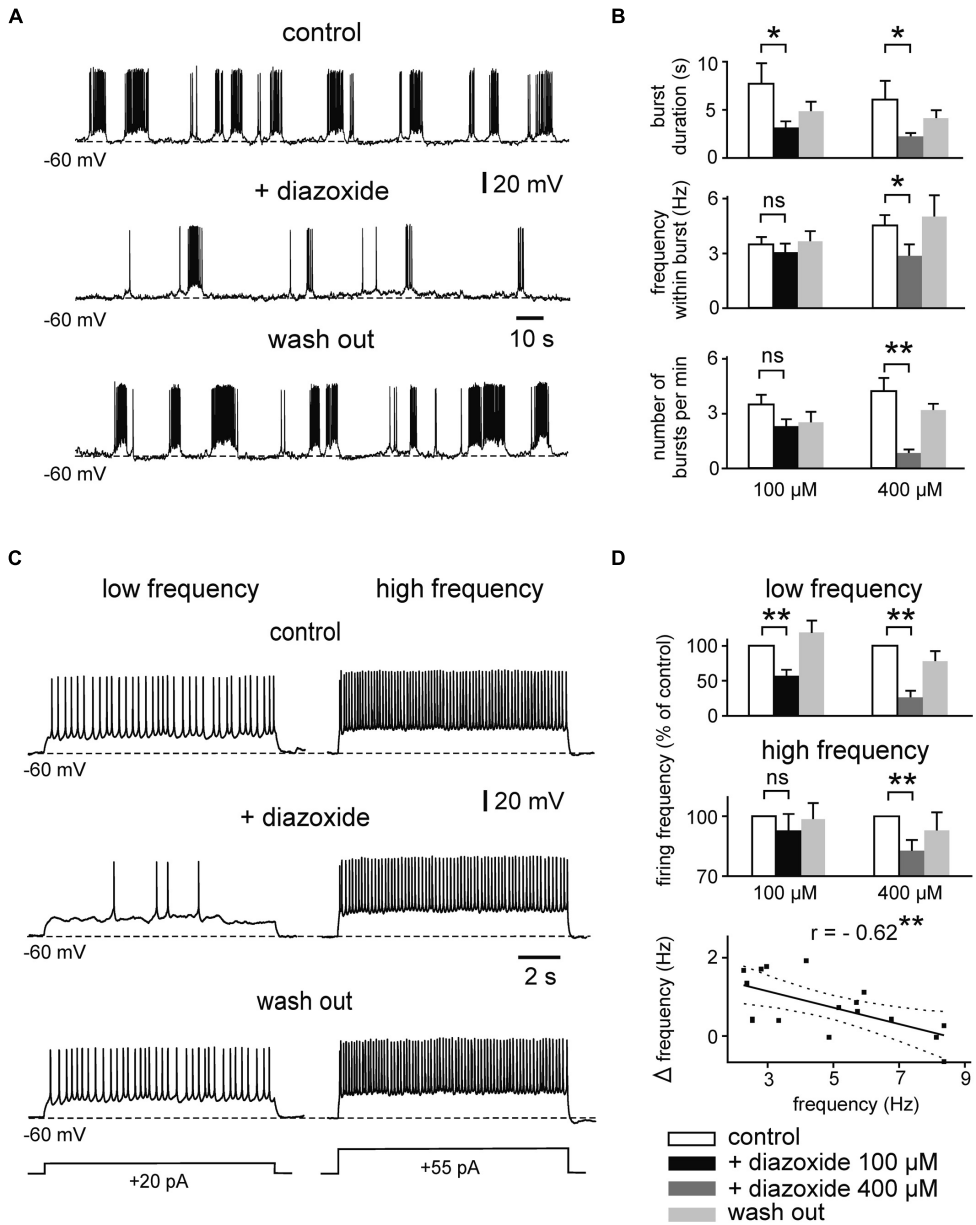
**Effects of the K_ATP_ channel opener diazoxide on bursting and regular firing activity of immature mEC layer III neurons. (A)** Example traces showing the reduction of prolonged bursts after activation of K_ATP_ channels by diazoxide (100 μM). **(B)** Mean effect of diazoxide on different burst parameters (left panel: 100 μM, *n* = 6; right panel: 400 μM, *n* = 8). White bars: control; black/dark gray: diazoxide; light gray: washout. **(C)** Activity-dependent effects of diazoxide (100 μM) on regular firing. A neuron firing at low rate (left) is more sensitive to diazoxide than the same cell firing at high frequency (right). **(D)** Bar diagrams showing normalized spike frequency under control conditions, in the presence of diazoxide (left panel 100 μM, *n* = 8; right panel 400 μM, *n* = 8 for low frequency, *n* = 6 for high frequency). Averaged data are given as mean ± SEM. Paired *t*-test or Wilcoxon test, *p < 0.05, **p < 0.01, ns, not significant. Bottom: correlation between basal firing frequency and diazoxide-mediated reduction of firing (Δfrequency = frequency in control - frequency under 100 μM diazoxide). Solid line indicates linear correlation (**two-tailed *p* < 0.01). Dashed lines indicate 95% confidence interval. Recordings were performed in the presence of kynurenic acid and picrotoxin and with pipette solution containing 4 mM ATP.

We next tested whether the intrinsic firing pattern of principal neurons in the immature mEC LIII is directly controlled by K_ATP_ channels. For these recordings, synaptic transmission was suppressed with kynurenic acid (2 mM) and picrotoxin (100 μM). We focused on the most prominent patterns of intrinsic neuronal firing activity at this stage, namely prolonged bursting and regular firing activity (Sheroziya et al., 2009). The percentage of bursting neurons depends on the extracellular concentration of Ca^2+^ (i.e., lowering [Ca^2+^]_o_ increases the tendency to generate bursts), and considerably changes during postnatal maturation. At 1 mM [Ca^2+^]_o,_ prolonged bursts are found in ∼50, 80, and 30% of LIII neurons at P5–P7, P8–P10, and P11–P13, respectively (Sheroziya et al., 2009). In a first set of experiments, we examined the effect of K_ATP_ channel openers on intrinsic firing. Whole-cell recordings were performed with standard intracellular solution containing 4 mM ATP. Resting membrane potential (RMP) of bursting neurons was -64.9 ± 0.9 mV (*n* = 29). In order to minimize effects of voltage on burst duration we manually adjusted membrane potential of all bursting neurons to ∼60 mV (-59.5 ± 0.4 mV, *n* = 29). Under these conditions, duration of prolonged bursts was 4.4 s (median, first quartile: 3.6 s and third quartile: 9.9 s, *n* = 29). The K_ATP_ channel opener diazoxide strongly suppressed prolonged bursting activity in LIII neurons (**Figures 2A,B**). At 100 μM diazoxide, burst duration was significantly decreased from 7.8 ± 2.1 s to 3.1 ± 0.8 s (*n* = 6, *p* = 0.031, Wilcoxon test). Action potential frequency within bursts and occurrence of bursts (i.e., number of bursts per minute) were slightly, but not significantly, reduced (control vs. diazoxide; 3.5 ± 0.4 vs. 3.0 ± 0.4 Hz for frequency within bursts, *p* = 0.380; 3.4 ± 0.4 vs. 2.3 ± 0.4 for number of bursts per minute, *p* = 0.073, *n* = 6, *t*-test for both parameters). However, at 400 μM, diazoxide significantly suppressed all of these parameters (*n* = 6; **Figure 2B**). Burst duration decreased from 6.2 ± 2.0 to 2.3 ± 0.4 s (*p* = 0.031, Wilcoxon test), frequency within bursts from 4.5 ± 0.6 Hz to 2.85 ± 0.65 Hz (*p* = 0.028, *t*-test), and number of bursts per minute from 4.2 ± 0.7 to 0.8 ± 0.2 (*p* = 0.006, *t*-test).

We next tested effects of diazoxide on regularly firing neurons, i.e., neurons which were silent at RMP (-63 ± 0.76 mV, *n* = 25) and showed regular spiking following membrane depolarization. The open state probability of K_ATP_ channels depends strongly on ATP concentration within submembrane domains which can vary depending on activity. We therefore performed experiments using depolarizing current pulses at different intensity (range 9–80 pA, duration 11 s) yielding “low” (∼1–3 Hz) and “high” (∼6–10 Hz) firing frequency, respectively. Indeed, as illustrated in **Figures 2C,D**, diazoxide (100 μM) supressed low frequency regular firing from 2.9 ± 0.2 to 1.7 ± 0.3 Hz (57%, *n* = 8, *p* = 0.002, *t*-test), while firing at higher frequencies was largely resistant to the drug (control vs. diazoxide; 6.8 ± 0.5 vs. 6.4 ± 0.7 Hz, *n* = 8, *p* = 0.075, *t*-test). At 400 μM, however, dizoxide reduced both, low and high frequency firing (control vs. diazoxide; 3.2 ± 0.4 vs. 0.9 ± 0.3 Hz, *n* = 8, *p* = 0.002, (low frequency) and 9.8 ± 0.9 vs. 8.2 ± 0.8 Hz (high frequency), *n* = 6, *p* = 0.001, *t*-test for both frequencies). In line with the activity-dependence of effects, reduction of firing frequency by the K_ATP_ opener diazoxide correlated negatively with control frequency (Pearson’s *r* = -0.62, *p* = 0.008; **Figure 2D**, bottom panel). RMP of regularly firing neurons was not significantly changed under diazoxide (*p* = 0.881 for 100 μM and *p* = 0.153 for 400 μM, *t*-test for both concentrations).

**FIGURE 3:**
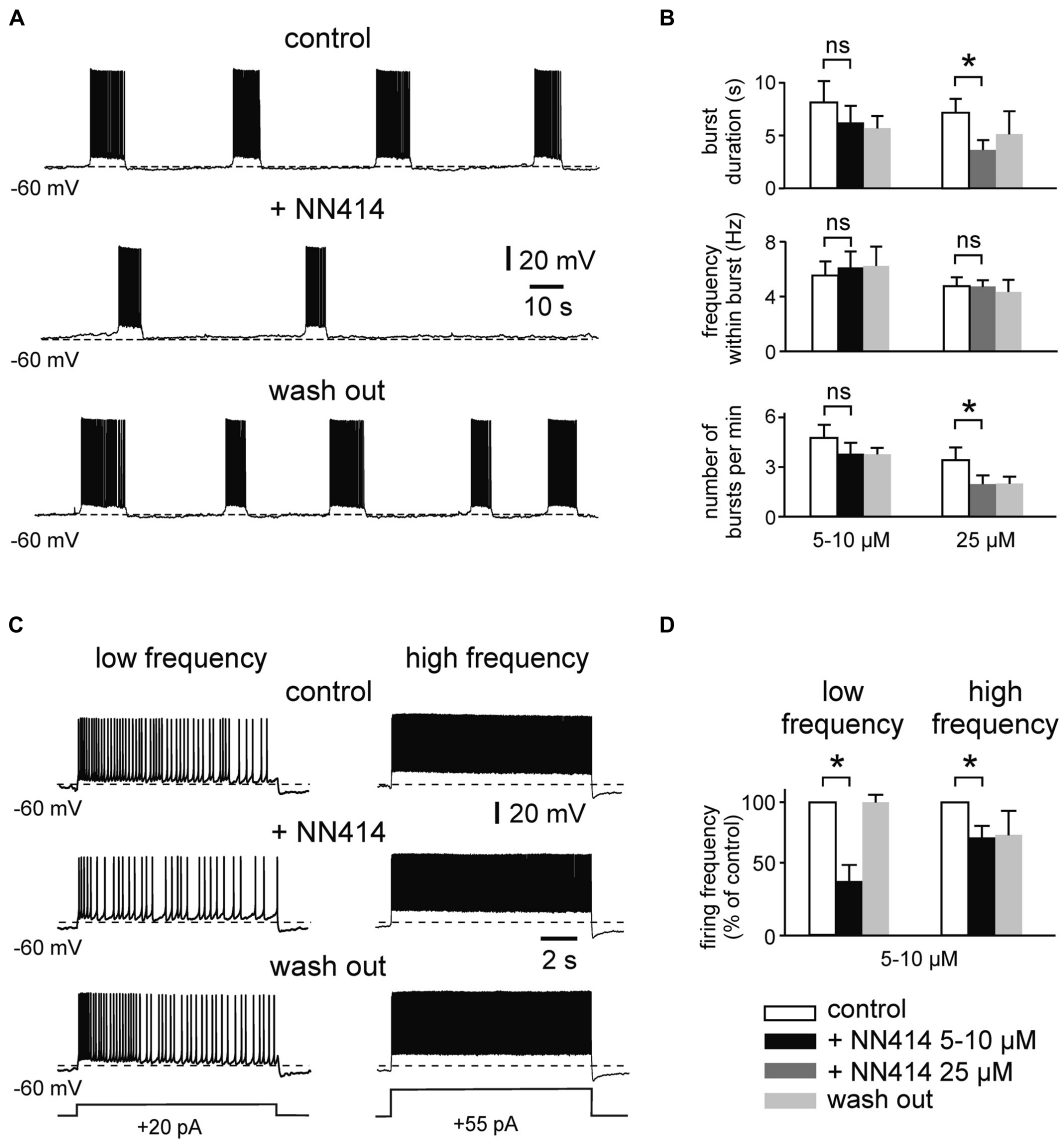
**The K_ATP_ channel opener NN414 suppresses intrinsic firing activity. (A)** Example trace showing suppression of prolonged bursting activity after activation of K_ATP_ channels with NN414 (25 μM). **(B)** Mean burst parameters under control conditions, in the presence of NN414 (left panel 5/10 μM, *n* = 8; right panel 25 μM, *n* = 7) and after wash out. **(C)** Effects of NN414 (5/10 μM) on regular spiking activity at low firing rate (left) and at high frequency (right; example traces from same cell). **(D)** Normalized regular firing frequency under control conditions, in the presence of NN414 (5/10 μM, *n* = 6) and after wash out. All recordings were made in the presence of kynurenic acid and picrotoxin. Patch pipette solution containing 4 mM ATP. Data are given as mean ± SEM. Paired *t*-test or Wilcoxon test, *p < 0.05, ns, not significant.

Similar effects were observed for the K_ATP_ channel opener NN414 (**Figure 3**). Thus, NN414 (25 μM) supressed burst duration from 7.1 ± 1.3 to 3.6 ± 0.9 s (*n* = 7, *p* = 0.015, *t*-test), and reduced the occurrence of bursts from 3.5 ± 0.8 to 2.0 ± 0.5 per minute (*n* = 7, *p* = 0.026, *t*-test; **Figures 3A,B**). Lower concentrations of NN414 (5–10 μM) induced apparent reductions of burst duration and burst occurrence, which were, however, not significant (*n* = 8, *p* = 0.174 and *p* = 0.480 respectively, *t*-test for both parameters; **Figure 3B**). In contrast, 5–10 μM of NN414 effectively reduced regular firing at low frequency (control vs. NN414; 3.1 ± 0.6 vs. 1.3 ± 0.5 Hz, *n* = 6, *p* = 0.013, *t*-test; **Figures 3C,D**). Higher firing frequency was slightly, but significantly reduced in the presence of the drug (control vs. NN414; 8.9 ± 1.0 vs. 6.6 ± 0.9 Hz, *n* = 6, *p* = 0.031, Wilcoxon test). In summary, these data suggest that K_ATP_ channel openers effectively suppress prolonged bursting and regular firing activity in immature mEC LIII neurons.

**FIGURE 4:**
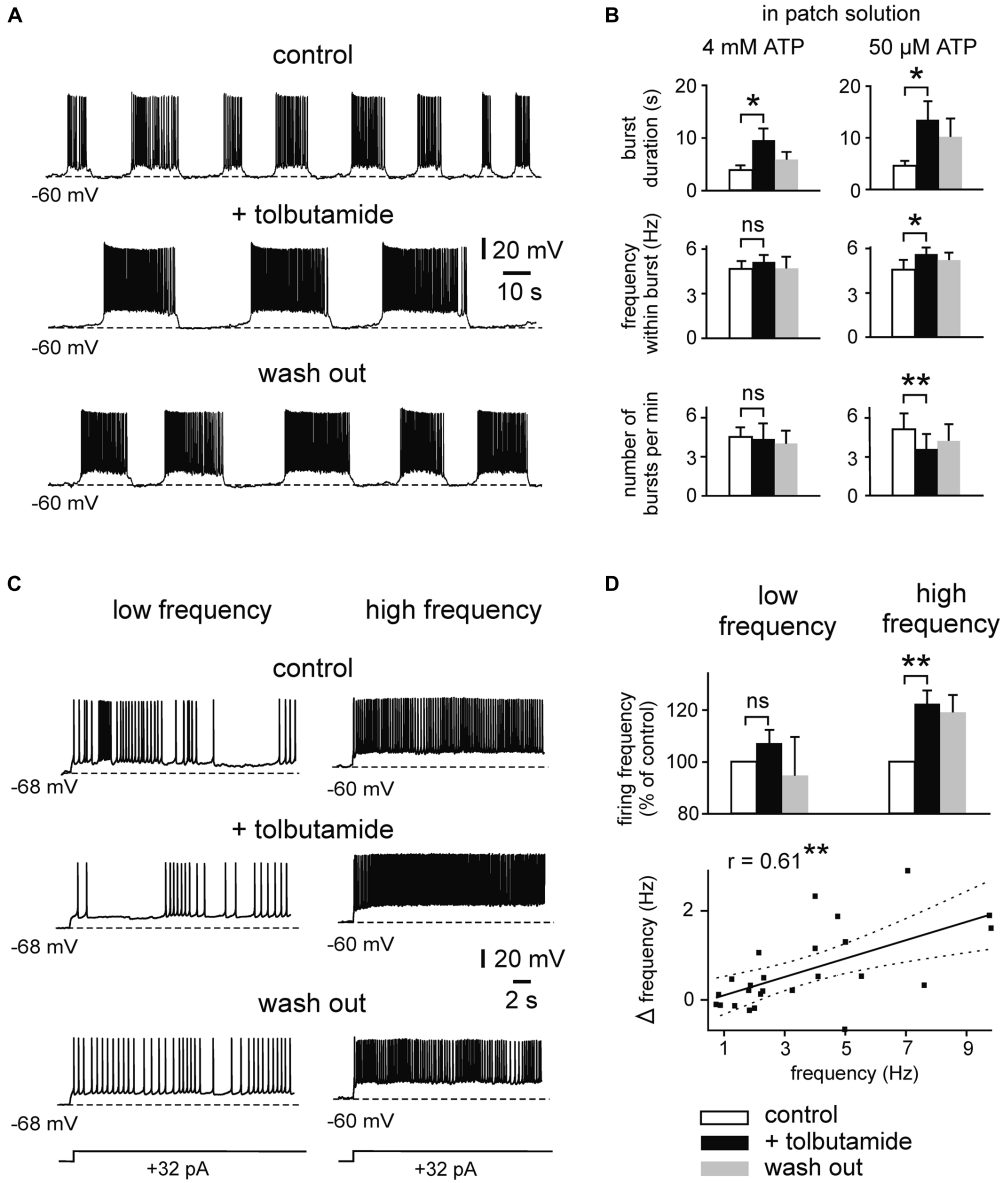
**Effects of the K_ATP_ channel blocker tolbutamide on intrinsic spiking activity. (A)** Example trace showing prolonged bursting activity under control conditions, in the presence of tolbutamide (300 μM) and after wash out. Recording was performed with pipette solution containing 50 μM ATP. **(B)** Mean burst parameters under control conditions, in the presence of tolbutamide and after wash out. Patch solutions containing 4 mM ATP (*n* = 4; left) and 50 μM (*n* = 7; right). **(C)** Example trace showing effects of tolbutamide (300 μM) on regular firing activity. At low firing rate (left) discharges are less sensitive to tolbutamide than at higher firing rate (right). **(D)** Normalized spike frequency under control conditions, in the presence of tolbutamide and after wash out (top panel; *n* = 12 for low frequency, *n* = 7 for high frequency). Averaged data are given as mean ± SEM. ANOVA followed by Dunn’s *post hoc* test (for B; 4 mM ATP) or Paired *t*-test, *p < 0.05, **p < 0.01, ns, not significant. Bottom: correlation between basal firing frequency and tolbutamide-mediated increase of firing (Δ frequency = frequency under 300 μM tolbutamide - control frequency). Solid line indicates linear correlation (**two-tailed *p* < 0.01). Dashed lines indicate 95% confidence interval. All recordings were obtained in the presence of kynurenic acid and picrotoxin.

**FIGURE 5:**
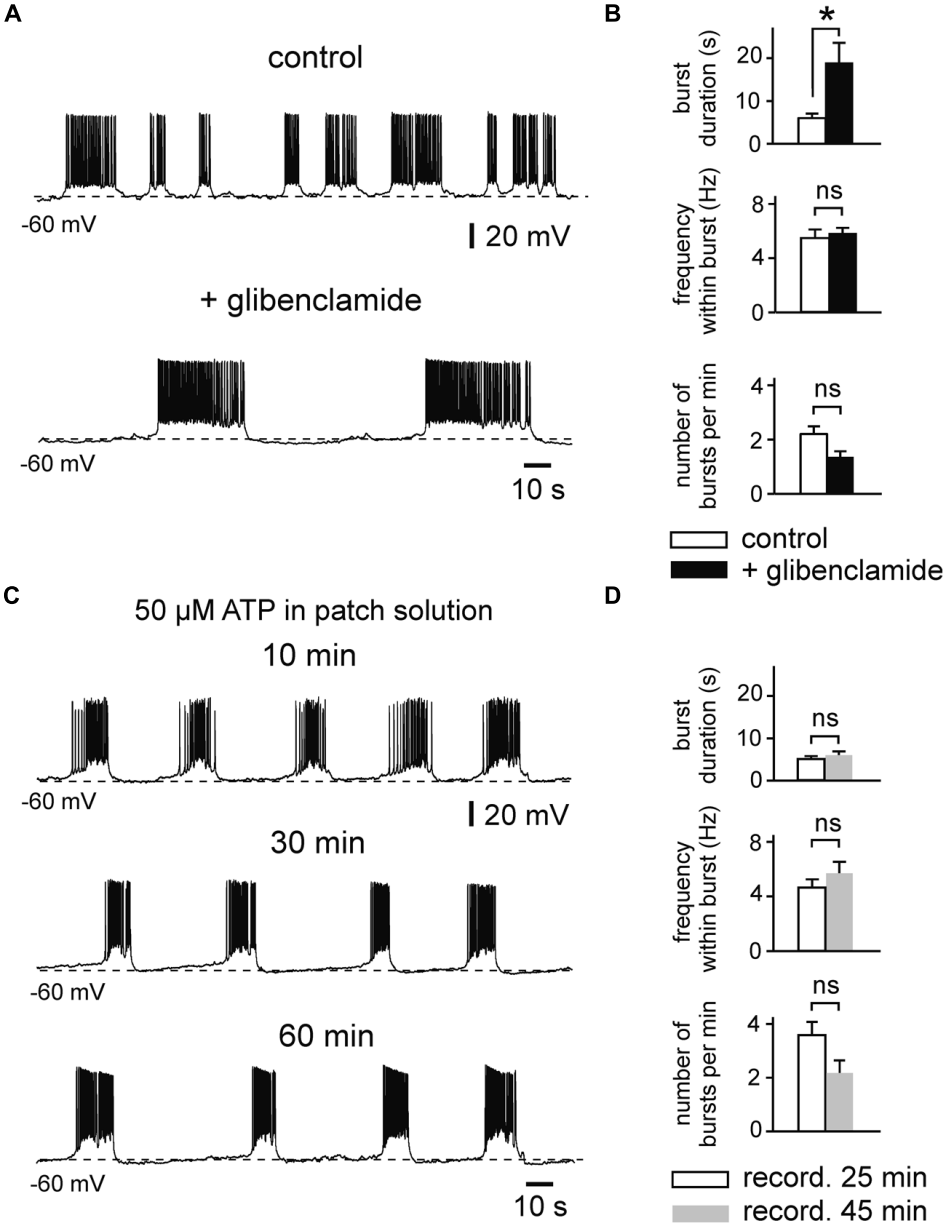
**Effects of the K_ATP_ channel blocker glibenclamide on prolonged bursting activity. (A)** Glibenclamide (1 μM) evokes prolongation of burst duration (example trace). **(B)** Changes of mean burst parameters by glibenclamide (*n* = 8). Patch pipette solution containing 50 μM ATP. **(C)** Example trace showing stable intrinsic bursting activity during prolonged whole-cell recording with pipette solution containing 50 μM ATP. **(D)** Burst parameters during prolonged recordings (*n* = 6). Note that there are no significant changes over 25–45 min. All recordings were obtained in the presence of kynurenic acid and picrotoxin. Averaged data are given as mean ± SEM. Paired *t*-test, *p < 0.05, ns, not significant.

We then investigated effects of K_ATP_ channel blockers on intrinsic firing activity (**Figures 4 and 5**). We first determined effects of tolbutamide (300 μM) on prolonged bursts using similar experimental conditions (4 mM ATP intracellularly). We found that tolbutamide extends burst duration from 3.8 ± 0.9 to 9.5 ± 2.3 s (*n* = 4; *p* < 0.05, ANOVA, Dunn’s *post hoc*), while frequency within bursts and number of bursts per minute were unaffected (*n* = 4, *p* > 0.05 for both parameters, ANOVA, Dunn’s *post hoc*; **Figure 4B**). ATP inhibits opening of K_ATP_ channels with an IC_50_ of ∼25 μM (Nichols et al., 1991). We therefore repeated our experiments with a lower intracellular concentration of ATP (50 μM) to avoid saturating effects on channel gating. Under these conditions RMP of bursting neurons was -67.6 ± 0.6 mV (*n* = 19) and RMP of regularly firing neurons was -65.5 ± 0.9 mV (*n* = 18). Membrane potential was manually adjusted to -61 ± 0.7 mV, resulting in spontaneous burst duration of 5.4 ± 0.5 s (*n* = 21). As illustrated in **Figures 4A,B**, tolbutamide (300 μM) significantly affected all tested parameters in experiments with reduced intracellular ATP concentration (control vs. tolbutamide; burst duration: 4.6 ± 1.0 vs. 13.5 ± 4.0 s, *p* = 0.030; frequency within burst: 4.6 ± 0.7 vs. 5.5 ± 0.7 Hz, *p* = 0.034; number of bursts per minute: 5.0 ± 1.3 vs. 3.6 ± 1.3, *p* = 0.004, *n* = 7, *t*-test for all values).

Likewise, tolbutamide increased firing frequency of regularly firing neurons when these were strongly activated (**Figures 4C,D**). Adding tolbutamide enhanced high frequency firing from 7.1 ± 0.8 to 8.6 ± 0.9 Hz (121%, *n* = 7, *p* = 0.004, *t*-test). However, tolbutamide was not effective at low frequency firing (control vs. tolbutamide; 1.8 ± 0.2 vs. 2.0 ± 0.3 Hz, *n* = 12, *p* = 0.095, *t*-test). In line with these activity-dependent effects, tolbutamide-mediated elevation of firing frequency correlated positively with control frequency (Pearson’s *r* = 0.61, *p* = 0.001; **Figure 4D**, bottom panel). RMP of regularly firing neurons was not affected by tolbutamide (*n* = 12, *p* = 0.397, *t*-test). In addition, in 4 out of 16 recording neurons, tolbutamide switched neuronal activity from regular firing into bursting mode. It has been reported that the widely used K_ATP_ channel blocker tolbutamide does also affect several Ca^2+^ – and voltage-dependent K^+^ currents in adult hippocampal neurons, including I_M_, I_AHP,_ and D-type K^+^ currents (Crépel et al., 1993; Erdemli and Krnjević, 1996). We therefore tested effects of a second K_ATP_ channel blocker, glibenclamide, on intrinsic bursts of immature LIII neurons (**Figures 5A,B**). Similar to tolbutamide, glibenclamide (1 μM) strongly enhanced duration of bursts (control vs. glibenclamide; 6.1 ± 0.9 vs. 19.0 ± 4.7 s, *n* = 8, *p* = 0.017, *t*-test), whereas no significant effects were found for frequency within bursts (5.5 ± 0.7 vs. 5.8 ± 0.5 Hz, *p* = 0.341, *t*-test) and for occurrence of bursts (2.2 ± 0.3 vs. 1.4 ± 0.2, *p* = 0.078, *t*-test). The effect of glibenclamide was, however, not reversible after washout of the drug. As a control, we therefore measured stability of burst parameters during prolonged recordings without drugs (50 μM ATP in the patch electrode). We did not find any significant changes during time-matched control recordings (*n* = 6, burst duration: *p* = 0.430, frequency within bursts: *p* = 0.101, number of bursts per minute: *p* = 0.119, *t*-test for all values; **Figures 5C,D**), indicating that the irreversible effects of glibenclamide were indeed specifically induced by the drug. These data show that K_ATP_ channel blockers efficiently enhance intrinsic firing activity. In summary, K_ATP_ channels exert powerful modulation of intrinsic bursting and regular firing activity as well as spontaneous early network oscillations in the immature mEC.

## DISCUSSION

We show that K_ATP_ channels are strongly involved in the modulation of spontaneous network oscillations and intrinsic firing activity in the immature rat mEC *in vitro.* Based on extracellular and intracellular recordings in the presence of K_ATP_-affecting drugs we report that these channels efficiently modulate electrical activity in layer III of the developing mEC.

K_ATP_ channel openers reduced the duration of spontaneous fp-bursts and suppressed intrinsic neuronal firing activity, including both prolonged bursting and regular firing patterns. In contrast, K_ATP_ channel blockers slightly prolonged fp-bursts and strongly enhanced the duration of intrinsic bursts as well as firing frequency of regularly firing neurons. In this latter case, effects at the cellular level seemed to be stronger than effects at the network (fp) level. Indeed, intrinsic bursts of single neurons contribute to, but not fully reflect the spontaneous fp-bursts, which arise from complex interactions between excitatory and inhibitory transmission as well as intrinsic neuronal properties. Termination of fp-bursts in immature EC is likely partly associated with activation of fast spiking GABAergic interneurons, as it has been reported for adult animals (Tahvildari et al., 2012). Indeed, a selective blockade of GABA_A_-receptors always elicited paroxysmal field discharges in the immature EC (Sheroziya et al., 2009). It is thus possible, that K_ATP_ channel blockers increase activity of fast spiking interneurons that restricts prolongation of fp-bursts.

Importantly, regular firing activity was modulated in a frequency-dependent manner, with strongest effects of the K_ATP_ channel blocker tolbutamide on highly active neurons, and strongest effects of the K_ATP_ channel opener diazoxide on slowly firing cells. These correlations might reflect transient changes in intracellular (submembrane) ATP concentration during electrical activity (Howarth et al., 2012). High-frequency firing activity might significantly reduce local ATP levels and, therefore, increase opening of K_ATP_ channels. In this situation, blockers of K_ATP_ are highly efficient while drugs increasing channel opening lose effect. At low firing frequencies, higher intracellular ATP levels would exert opposite effects.

Prolonged intrinsic bursting activity is a characteristic feature of developing, but not mature, rat mEC LIII neurons (Sheroziya et al., 2009). The ionic mechanisms underlying the generation of burst firing include activation of various currents: Ca^2+^-sensitive non-specific cationic current (I_CAN_), persistent Na^+^ current (I_Nap_) and Ca^2+^-activated K^+^ current (I_AHP_, BK-current; Sheroziya and Egorov, 2008; Sheroziya et al., 2009). Our present data show that ATP-sensitive K^+^ current is also involved in the generation of prolonged intrinsic bursts. Number and frequency of spikes during prolonged bursts appear to be more than enough to induce opening of K_ATP_ channels, that, together with I_AHP_, finally mediate burst termination.

Effects of the K_ATP_ channel blocker tolbutamide were prominent at low intracellular concentrations of ATP (50 μM). However, the drug was effective even at high ATP concentrations in the patch electrode (4 mM), as frequently used in whole-cell recordings. This value is much higher than the half*-*maximal inhibitory concentration of ATP for K_ATP_ channels (∼25 μM; Nichols et al., 1991). Thus, our data support the proposal that open probability of K_ATP_ channels reflects activity-dependent fluctuations of ATP/ADP concentrations within local submembrane domains which are not entirely controlled by the solution in the patch pipette (Haller et al., 2001; Mollajew et al., 2013). It is likely that such local changes in ADP/ATP ratio represent submembrane ATP consumption during increased Na^+^-K^+^-ATPase activity (Haller et al., 2001).

In the adult rat hippocampus, the K_ATP_ channel blocker tolbutamide also affects several Ca^2+^ – and voltage-dependent K^+^ currents, including M-type K^+^ current and I_AHP_ (Crépel et al., 1993; Erdemli and Krnjević, 1996). We can not exclude that such mechanisms do also play some role in the immature mEC. It has been reported, however, that K_ATP_ channels themselves participate in the slow afterhyperpolarization (sAHP) in hippocampal dentate granule cells of juvenile mice, and thereby affect neuronal excitability following elevated firing (Tanner et al., 2011). The role of M-currents in immature neuronal excitability seems to be not significant. For example, the Kv7/M channel blocker linopirdine has only a minor effect on neonatal activity, in contrast to juvenile CA3 pyramidal neurons (Safiulina et al., 2008).

In addition, the hyperpolarization-activated current (Ih) plays an important role in modulation of excitability of adult LIII neurons (Shah et al., 2004; Huang et al., 2009). Altered expression of Ih underlying hyperpolarization-activated cyclic nucleotide-gated (HCN) channel subunits has been reported from models of temporal lobe epilepsy where it affects seizure threshold (Shah et al., 2004). Expression of HCN1 subunits in EC LIII neurons is, however, dominant in distal dendrites, and is quite moderate in somata of these cells, particular at early stages (Vasilyev and Barish, 2002). Nevertheless, we do not exclude a contribution of Ih to prolonged burst firing of immature LIII neurons.

Our results are also in line with previous reports showing that K_ATP_ channel can be activated in response to ATP consumption during normal (i.e., physiological) levels of neuronal activity. The open state probability of K_ATP_ channel augments in response to ATP consumption during moderate neuronal firing (Haller et al., 2001; Tanner et al., 2011) or via prolonged activation of glutamate receptors (Mollajew et al., 2013). K_ATP_ channel are strongly involved in the modulation of various neurophysiological functions. In dopaminergic neurons of the medial substantia nigra, activity of K_ATP_ channels enables burst firing *in vitro* and *in vivo*, thereby controlling novelty-induced exploratory behavior (Schiemann et al., 2012). In hypothalamic neurons expressing pro-opiomelanocortin (POMC) the age-dependent up-regulation of K_ATP_ channels causes hyperpolarization and neuronal silencing, contributing to obesity of aged animals (Yang et al., 2012). K_ATP_ channel are also involved in pathologically altered network activity such as epileptic seizures (Hernández-Sánchez et al., 2001; Yamada et al., 2001).

In the immature EC, spontaneous fps and large-scale propagating oscillatory calcium transients mediated by ionotropic glutamate (but not GABA) receptors have been reported (Jones and Heinemann, 1989; Garaschuk et al., 2000; Sheroziya et al., 2009; Namiki et al., 2013). At mature stages, mEC neurons can generate slow-wave network oscillations (Dickson et al., 2003), which are initiated by selective activation of GluR5 kainate receptors in a recurrent network, and terminated by activation of K_ATP_ channels in active neurons (Cunningham et al., 2006). This example illustrates the tight coupling between neuronal activity and energy homeostasis. In the immature mEC, however, network oscillations are generated by different mechanisms, including the joined activation of both NMDA and AMPA/kainate receptors (Sheroziya et al., 2009). Moreover, in contrast to the observations in adult rats, in our hands the K_ATP_ channel opener diazoxide suppressed, but never completely blocked fp-bursts. This finding indicates a differential impact of ATP-sensitive K^+^ currents on immature network activity versus mature sleep-related slow-wave oscillations in the mEC. Importantly, it has been shown that LIII principal neurons are critically involved in the processes of initiation, propagation, termination, and reflection of synchronized traveling calcium waves in the immature EC (Namiki et al., 2013). This mechanism may be related to the ability of these neurons to generate prolonged intrinsic bursting activity at early postnatal stages.

The functional significance of prolonged intrinsic bursts in immature EC networks are still unknown. They may well contribute to the functional maturation of the EC and anatomically connected areas. Recurrent excitatory connections and electrical coupling between EC LIII pyramidal neurons have been demonstrated in adult rats using paired intracellular recordings (Dhillon and Jones, 2000). Intralaminar excitatory recurrent connections within LIII of juvenile rats have been recently shown using scanning photostimulation (Beed et al., 2010). Thus, activity of immature LIII neurons feeds back onto neurons within the same layer, and therefore may contribute to the activity-dependent maturation of this local network in the EC. In addition, EC LIII neurons provide direct input to the apical dendrites of CA1 pyramids. Therefore, bursts of LIII neurons may induce dendritic spikes in CA1 pyramidal neurons that could then propagate to the soma and trigger action potentials (Jarsky et al., 2005). Strong firing of LIII neurons can also contribute to the distal dendritic enrichment of HCN1 channels in CA1 pyramids (Shin and Chetkovich, 2007). In adult animals, excitatory input from EC LIII to CA1 is essential for the formation of temporal association memory and fear learning (Suh et al., 2011; Kitamura et al., 2014; Lovett-Barron et al., 2014).

To conclude, our results show that activation of K_ATP_ channels plays an important, limiting role for network activity and intrinsic neuronal firing in the immature mEC. This finding provides new insights into the mechanisms of activity-dependent maturation of neurons and networks in the EC. In addition, the key role of K_ATP_ channels constitutes an important link between neuronal activity and neurometabolic state in this important area of the medial temporal lobe.

## Conflict of Interest Statement

The authors declare that the research was conducted in the absence of any commercial or financial relationships that could be construed as a potential conflict of interest.

## References

[B1] Aguilar-Bryan L. Bryan J. (1999). Molecular biology of adenosine triphosphate-sensitive potassium channels. *Endocr. Rev.* 20 101–135 10204114 10.1210/edrv.20.2.0361

[B2] Allène C. Cattani A. Ackman J. B. Bonifazi P. Aniksztejn L. Ben-Ari Y. (2008). Sequential generation of two distinct synapse-driven network patterns in developing neocortex. *J. Neurosci.* 28 12851–12863 10.1523/JNEUROSCI.3733-08.2008 19036979 PMC6671804

[B3] Ashcroft S. J. Ashcroft F. M. (1990). Properties and functions of ATP-sensitive K-channels. *Cell. Signal.* 2 197–214 10.1016/0898-6568(90)90048-F 2119205

[B4] Beed P. Bendels M. H. Wiegand H. F. Leibold C. Johenning F. W. Schmitz D. (2010). Analysis of excitatory microcircuitry in the medial entorhinal cortex reveals cell-type-specific differences. *Neuron* 68 1059–1066 10.1016/j.neuron.2010.12.009 21172609

[B5] Ben-Ari Y. (2002). Excitatory actions of gaba during development: the nature of the nurture. *Nat. Rev. Neurosci.* 3 728–739 10.1038/nrn920 12209121

[B6] Buzsáki G. Moser E. I. (2013). Memory, navigation and theta rhythm in the hippocampal-entorhinal system. *Nat. Neurosci.* 16 130–138 10.1038/nn.3304 23354386 PMC4079500

[B7] Crepel V. Aronov D. Jorquera I. Represa A. Ben-Ari Y. Cossart R. (2007). A parturition-associated nonsynaptic coherent activity pat tern in the developing hippocampus. *Neuron* 54 105–120 10.1016/j.neuron.2007.03.007 17408581

[B8] Crépel V. Krnjević K. Ben-Ari Y. (1993). Sulphonylureas reduce the slowly inactivating D-type outward current in rat hippocampal neurons. *J. Physiol.* 466 39–54 8410699 PMC1175465

[B9] Cunningham M. O. Pervouchine D. D. Racca C. Kopell N. J. Davies C. H. Jones R. S. (2006). Neuronal metabolism governs cortical network response state. *Proc. Natl. Acad. Sci. U.S.A.* 103 5597–5601 10.1073/pnas.0600604103 16565217 PMC1459399

[B10] Dabrowski M. Larsen T. Ashcroft F. M. Bondo Hansen J. Wahl P. (2003). Potent and selective activation of the pancreatic beta-cell type KATP channel by two novel diazoxide analogues. *Diabetologia* 46 1375–1382 10.1007/s00125-003-1198-1 12961066

[B11] Dhillon A. Jones R. S. (2000). Laminar differences in recurrent excitatory transmission in the rat entorhinal cortex in vitro. *Neuroscience* 99 413–422 10.1016/S0306-4522(00)00225-6 11029534

[B12] Dickson C. T. Biella G. de Curtis M. (2003). Slow periodic events and their transition to gamma oscillations in the entorhinal cortex of the isolated Guinea pig brain. *J. Neurophysiol.* 90 39–46 10.1152/jn.01063.2002 12843303

[B13] Dickson C. T. Mena A. R. Alonso A. (1997). Electroresponsiveness of medial entorhinal cortex layer III neurons in vitro. *Neuroscience* 81 937–950 10.1016/S0306-4522(97)00263-7 9330357

[B14] Dunn-Meynell A. A. Rawson N. E. Levin B. E. (1998). Distribution and phenotype of neurons containing the ATP-sensitive K^+^ channel in rat brain. *Brain Res.* 14 41–54 10.1016/S0006-8993(98)00956-1 9838037

[B15] Dupont E. Hanganu I. L. Kilb W. Hirsch S. Luhmann H. J. (2006). Rapid developmental switch in the mechanisms driving early cortical columnar networks. *Nature* 439 79–83 10.1038/nature04264 16327778

[B16] Egorov A. V. Draguhn A. (2013). Development of coherent neuronal activity patterns in mammalian cortical networks: common principles and local hetereogeneity. *Mech. Dev.* 130 412–423 10.1016/j.mod.2012.09.006 23032193

[B17] Erdemli G. Krnjević K. (1996). Tolbutamide blocks Ca^(2+)^- and voltage-dependent K^+^ currents of hippocampal CA1 neurons. *Eur. J. Pharmacol.* 304 37–47 10.1016/0014-2999(96)00124-0 8813582

[B18] Erecinska M. Cherian S. Silver I. A. (2004). Energy metabolism in mammalian brain during development. *Prog. Neurobiol.* 73 397–445 10.1016/j.pneurobio.2004.06.003 15313334

[B19] Feller M. B. Wellis D. P. Stellwagen D. Werblin F. S. Shatz C. J. (1996). Requirement for cholinergic synaptic transmission in the propagation of spontaneous retinal waves. *Science* 272 1182–1187 10.1126/science.272.5265.1182 8638165

[B20] Fujimura N. Tanaka E. Yamamoto S. Shigemori M. Higashi H. (1997). Contribution of ATP-sensitive potassium channels to hypoxic hyperpolarization in rat hippocampal CA1 neurons in vitro. *J. Neurophysiol.* 77 378–385 9120578 10.1152/jn.1997.77.1.378

[B21] Garaschuk O. Linn J. Eilers J. Konnerth A. (2000). Large-scale oscillatory calcium waves in the immature cortex. *Nat. Neurosci.* 3 452–459 10.1038/74823 10769384

[B22] Gloveli T. Schmitz D. Empson R. M. Dugladze T. Heinemann U. (1997). Morphological and electrophysiological characterization of layer III cells of the medial entorhinal cortex of the rat. *Neuroscience* 77 629–648 10.1016/S0306-4522(96)00494-0 9070741

[B23] Haller M. Mironov S. L. Karschin A. Richter D. W. (2001). Dynamic activation of K(ATP) channels in rhythmically active neurons. *J. Physiol.* 15 69–81 10.1111/j.1469-7793.2001.0069k.x 11711562 PMC2278932

[B24] Hernández-Sánchez C. Basile A. S. Fedorova I. Arima H. Stannard B. Fernandez A. M. (2001). Mice transgenically overexpressing sulfonylurea receptor 1 in forebrain resist seizure induction and excitotoxic neuron death. *Proc. Natl. Acad. Sci. U.S.A.* 98 3549–3554 10.1073/pnas.051012898 11248115 PMC30690

[B25] Howarth C. Gleeson P. Attwell D. (2012). Updated energy budgets for neural computation in the neocortex and cerebellum. *J. Cereb. Blood Flow Metab.* 32 1222–1232 10.1038/jcbfm.2012.35 22434069 PMC3390818

[B26] Huang Z. Walker M. C. Shah M. M. (2009). Loss of dendritic HCN1 subunits enhances cortical excitability and epileptogenesis. *J. Neurosci.* 29 10979–10988 10.1523/JNEUROSCI.1531-09.2009 19726656 PMC2744118

[B27] Jarsky T. Roxin A. Kath W. L. Spruston N. (2005). Conditional dendritic spike propagation following distal synaptic activation of hippocampal CA1 pyramidal neurons. *Nat. Neurosci.* 12 1667–1676 10.1038/nn1599 16299501

[B28] Jones R. S. Heinemann U. (1989). Spontaneous activity mediated by NMDA receptors in immature rat entorhinal cortex in vitro. *Neurosci. Lett.* 104 93–98 10.1016/0304-3940(89)90335-2 2573017

[B29] Kandler K. Katz L. C. (1998). Coordination of neuronal activity in developing visual cortex by gap junction-mediated biochemical communication. *J. Neurosci.* 18 1419–1427 9454851 10.1523/JNEUROSCI.18-04-01419.1998 PMC6792729

[B30] Karschin C. Ecke C. Ashcroft F. M. Karschin A. (1997). Overlapping distribution of K(ATP) channel-forming Kir6.2 subunit and the sulfonylurea receptor SUR1 in rodent brain. *FEBS Lett.* 401 59–64 10.1016/S0014-5793(96)01438-X 9003806

[B31] Kasyanov A. M. Safiulina V. F. Voronin L. L. Cherubini E. (2004). GABA-mediated giant depolarizing potentials as coincidence detectors for enhancing synaptic efficacy in the developing hippocampus. *Proc. Natl. Acad. Sci. U.S.A.* 101 3967–3972 10.1073/pnas.0305974101 15007179 PMC374353

[B32] Khazipov R. Luhmann H. J. (2006). Early patterns of electrical activity in the developing cerebral cortex of humans and rodents. *Trends Neurosci.* 29 414–418 10.1016/j.tins.2006.05.007 16713634

[B33] Kitamura T. Pignatelli M. Suh J. Kohara K. Yoshiki A. Abe K. (2014). Island cells control temporal association memory. *Science* 343 896–901 10.1126/science.1244634 24457215 PMC5572219

[B34] Leinekugel X. Medina I. Khalilov I. Ben-Ari Y. Khazipov R. (1997). Ca^2+^ oscillations mediated by the synergistic excitatory action of GABA_A_ and NMDA receptors in the neonatal hippocampus. *Neuron* 18 243–255 10.1016/S0896-6273(00)80265-2 9052795

[B35] Lovett-Barron M. Kaifosh P. Kheirbek M. A. Danielson N. Zaremba J. D. Reardon T. R. (2014). Dendritic inhibition in the hippocampus supports fear learning. *Science* 343 857–863 10.1126/science.1247485 24558155 PMC4018419

[B36] Mohajerani M. H. Cherubini E. (2006). Role of giant depolarizing potentials in shaping synaptic currents in the developing hippocampus. *Crit. Rev. Neurobiol.* 18 13–23 10.1615/CritRevNeurobiol.v18.i1-2.30 17725505

[B37] Mollajew R. Toloe J. Mironov S. L. (2013). Single KATP channel opening in response to stimulation of AMPA/kainate receptors is mediated by Na^+^ accumulation and submembrane ATP and ADP changes. *J. Physiol.* 591 2593–2609 10.1113/jphysiol.2012.248369 23507878 PMC3678045

[B38] Namiki S. Norimoto H. Kobayashi C. Nakatani K. Matsuki N. Ikegaya Y. (2013). Layer III neurons control synchronized waves in the immature cerebral cortex. *J. Neurosci.* 33 987–1001 10.1523/JNEUROSCI.2522-12.2013 23325237 PMC6704853

[B39] Nelson K. B. (1989). Relationship of intrapartum and delivery room events to long-term neurologic outcome. *Clin. Perinatol.* 16 995–1007 2686900

[B40] Nichols C. G. (2006). KATP channels as molecular sensors of cellular metabolism. *Nature* 440 470–476 10.1038/nature04711 16554807

[B41] Nichols C. G. Lederer W. J. Cannell M. B. (1991). ATP dependence of KATP channel kinetics in isolated membrane patches from rat ventricle. *Biophys. J.* 5 1164–1177 10.1016/S0006-3495(91)82152-X 1760506 PMC1260172

[B42] Safiulina V. F. Zacchi P. Taglialatela M. Yaari Y. Cherubini E. (2008). Low expression of Kv7/M channels facilitates intrinsic and network bursting in the developing rat hippocampus. *J. Physiol.* 586 5437–5453 10.1113/jphysiol.2008.156257 18801845 PMC2655386

[B43] Schiemann J. Schlaudraff F. Klose V. Bingmer M. Seino S. Magill P. J. (2012). K-ATP channels in dopamine substantia nigra neurons control bursting and novelty-induced exploration. *Nat. Neurosci.* 15 1272–1280 10.1038/nn.3185 22902720 PMC4242970

[B44] Seino S. (1999). ATP-sensitive potassium channels: a model of heteromultimeric potassium channel/receptor assemblies. *Annu. Rev. Physiol.* 61 337–362 10.1146/annurev.physiol.61.1.337 10099692

[B45] Shah M. M. Anderson A. E. Leung V. Lin X. Johnston D. (2004). Seizure-induced plasticity of h channels in entorhinal cortical layer III pyramidal neurons. *Neuron* 44 495–508 10.1016/j.neuron.2004.10.011 15504329 PMC2386958

[B46] Sheroziya M. G. Egorov A. V. (2008). Effect of extracellular calcium on intrinsically bursting neurons in the entorhinal cortex of newborn rats: the computer modeling study. *Zh. Vyssh. Nerv. Deiat. Im. I P Pavlova* 58 517–520 19004312

[B47] Sheroziya M. G. von Bohlen und Halbach O. Unsicker K. Egorov A. V. (2009). Spontaneous bursting activity in the developing entorhinal cortex. *J. Neurosci.* 29 12131–12144 10.1523/JNEUROSCI.1333-09.2009 19793971 PMC6666150

[B48] Shin M. Chetkovich D. M. (2007). Activity-dependent regulation of h channel distribution in hippocampal CA1 pyramidal neurons. *J. Biol. Chem.* 282 33168–33180 10.1074/jbc.M703736200 17848552 PMC2685032

[B49] Sipilä S. T. Huttu K. Soltesz I. Voipio J. Kaila K. (2005). Depolarizing GABA acts on intrinsically bursting pyramidal neurons to drive giant depolarizing potentials in the immature hippocampus. *J. Neurosci.* 25 5280–5289 10.1523/JNEUROSCI.0378-05.2005 15930375 PMC6725004

[B50] Squire L. R. Stark C. E. Clark R. E. (2004). The medial temporal lobe. *Annu. Rev. Neurosci.* 27 279–306 10.1146/annurev.neuro.27.070203.144130 15217334

[B51] Suh J. Rivest A. J. Nakashiba T. Tominaga T. Tonegawa S. (2011). Entorhinal cortex layer III input to the hippocampus is crucial for temporal association memory. *Science* 334 1415–1420 10.1126/science.1210125 22052975

[B52] Sun H. S. Feng Z. P. Barber P. A. Buchan A. M. French R. J. (2007). Kir6.2-containing ATP-sensitive potassium channels protect cortical neurons from ischemic/anoxic injury in vitro and in vivo. *Neuroscience* 144 1509–1515 10.1016/j.neuroscience.2006.10.043 17175112

[B53] Tahvildari B. Wölfel M. Duque A. McCormick D. A. (2012). Selective functional interactions between excitatory and inhibitory cortical neurons and differential contribution to persistent activity of the slow oscillation. *J. Neurosci.* 32 12165–12179 10.1523/JNEUROSCI.1181-12.2012 22933799 PMC3466092

[B54] Tanner G. R. Lutas A. Martínez-François J. R. Yellen G. (2011). Single K ATP channel opening in response to action potential firing in mouse dentate granule neurons. *J. Neurosci.* 31 8689–8696 10.1523/JNEUROSCI.5951-10.2011 21653873 PMC3133530

[B55] van Strien N. M. Cappaert N. L. Witter M. P. (2009). The anatomy of memory: an interactive overview of the parahippocampal-hippocampal network. *Nat. Rev. Neurosci.* 10 272–282 10.1038/nrn2614 19300446

[B56] Vasilyev D. V. Barish M. E. (2002). Postnatal development of the hyperpolarization-activated excitatory current Ih in mouse hippocampal pyramidal neurons. *J. Neurosci.* 22 8992–9004 12388606 10.1523/JNEUROSCI.22-20-08992.2002 PMC6757670

[B57] Witter M. P. Amaral D. G. (2004). “Hippocampal formation,” in *The Rat Nervous System* 3rd Edn ed.Paxinos G. T. (San Diego: Academic Press) 635–704

[B58] Yamada K. Ji J. J. Yuan H. Miki T. Sato S. Horimoto N. (2001). Protective role of ATP-sensitive potassium channels in hypoxia-induced generalized seizure. *Science* 292 1543–1546 10.1126/science.1059829 11375491

[B59] Yang S. B. Tien A. C. Boddupalli G. Xu A. W. Jan Y. N. Jan L. Y. (2012). Rapamycin ameliorates age-dependent obesity associated with increased mTOR signaling in hypothalamic POMC neurons. *Neuron* 75 425–436 10.1016/j.neuron.2012.03.043 22884327 PMC3467009

[B60] Yoshida M. Alonso A. A. (2007). Cell-type specific modulation of intrinsic firing properties and subthreshold membrane oscillations by the M(Kv7)-current in neurons of the entorhinal cortex. *J. Neurophysiol.* 98 2779–2794 10.1152/jn.00033.2007 17728392

[B61] Zawar C. Plant T. D. Schirra C. Konnerth A. Neumcke B. (1999). Cell-type specific expression of ATP-sensitive potassium channels in the rat hippocampus. *J. Physiol.* 514 327–341 9852317 10.1111/j.1469-7793.1999.315ae.x PMC2269073

[B62] Zheng J. Lee S. Zhou Z. J. (2006). A transient network of intrinsically bursting starburst cells underlies the generation of retinal waves. *Nat. Neurosci.* 9 363–371 10.1038/nn1644 16462736

